# LncRNA Gm14205 induces astrocytic NLRP3 inflammasome activation via inhibiting oxytocin receptor in postpartum depression

**DOI:** 10.1042/BSR20200672

**Published:** 2020-08-07

**Authors:** Jialei Zhu, Jing Tang

**Affiliations:** Obstetrics and Gynecology Hospital of Fudan University, 419 Fangxie Road, Huangpu District, Shanghai 200011, China

**Keywords:** Astrocyte, Long non-coding RNA, NLRP3 inflammasome, Oxytocin receptor, Postpartum depression

## Abstract

Postpartum depression (PPD) is a kind of mental disorder characterized by persistent low emotions in puerperium. The most significant physiological change in postpartum is lactation which is regulated by oxytocin receptor (OXTR). However, whether OXTR is related to pathological process of PPD and the potential mechanism still remain unclear. In the present study, we prepared hormone-simulated pregnancy (HSP)-induced PPD mouse model and found that the protein level of OXTR in hippocampus of PPD model mice was down-regulated and Nod-like receptor protein 3 (NLRP3) inflammasome was activated. We identified five long non-coding RNAs (lncRNAs) related to PPD by transcriptome sequencing, including three up-regulated and two down-regulated. The five lncRNAs were associated with the signaling pathway of OXTR according to the bioinformatics analysis. Furthermore, we focused on one of the five lncRNAs, Gm14205, and found that it targeted OXTR which inhibited astrocytic NLRP3 inflammasome activation in hippocampal primary astrocytes. These findings illustrate that OXTR has protective effects in PPD by inhibiting NLRP3 inflammasome activation and provides a new strategy for targeting lncRNA Gm14205 in the pathogenesis of PPD.

## Introduction

Postpartum depression (PPD) is a mental disorder characterized by persistent depression in puerperium [1,2]. The main manifestations are depression, insomnia, anxiety, sadness, guilt, irritability and even suicidal tendency [3–5]. It usually attacks in the weeks after delivery and disappears within half a year, but serious cases can last for 1–2 years [6,7]. The pathological mechanism of PPD is still unclear [3,8–10]. PPD not only affects physical and mental health of puerperas, but also deprives infants of the effective care from their mothers, and affects marriage relations [4,11]. Therefore, it is important and urgent to explore the pathological mechanisms of PPD and search for the key target of its development.

The most significant physiological change in postpartum is lactation, and oxytocin is a key molecule mainly secreted in paraventricular nucleus and supraoptic nucleus of hypothalamus that contributes to lactation [12]. It participates in the regulation of cognition, social behavior, addiction [13,14], and also plays an important role in the treatment of psychiatric diseases [15]. Oxytocin exerts physiological functions by binding to oxytocin receptor (OXTR) [16]. OXTRs distribute mainly in hippocampus, hypothalamus, nucleus accumbens and also in uterine smooth muscle cells, vascular endothelial cells, adipocytes [17]. The number of OXTRs in different brain regions is closely related to the social behavior [18,19]. It has been reported that hypermethylation [20,21] and low expression [22] of OXTR may play important roles in the etiology of PPD susceptible phenotypes, suggesting that the post-transcriptional mechanisms may regulate the occurrence of PPD. Therefore, the study of the regulation of OXTR by non-coding RNA may help us understand the mechanism of PPD more deeply.

Long non-coding RNA (lncRNA) is a non-coding RNA whose length exceeds 200 nt. It plays an important role in epigenetics, DNA methylation, gene silencing or activation, transcription and post-transcriptional regulation [23–25]. Studies have shown that lncRNA may be involved in the pathophysiological process of depression [26]. However, the regulation of lncRNA on PPD, such a special type of depression, has not been reported yet. The scientific significance and applicability of lncRNA in clinical diagnosis and treatment of PPD need to be revealed.

In the present study, we prepared hormone-simulated pregnancy (HSP)-induced mouse model of PPD so as to investigate whether lncRNAs regulate PPD by targeting OXTR. We showed that the protein level of OXTR in hippocampus of PPD model mice was down-regulated and Nod-like receptor protein 3 (NLRP3) inflammasome was activated. We identified five lncRNAs related to PPD by transcriptome sequencing, including three up-regulated and two down-regulated. The five lncRNAs are associated with the signaling pathway of OXTR according to the bioinformatics analysis. Furthermore, we focused on one of the five lncRNAs, Gm14205, and found that it targeted OXTR which inhibited astrocytic NLRP3 inflammasome activation *in vitro*. These findings illustrate that OXTR has protective effects in PPD and provides a new strategy for targeting the lncRNA in the pathogenesis of this disease.

## Materials and methods

### Animals

C57BL/6J mice (female, 3-month-old) were purchased from SipprBK Laboratory Animals Ltd (Shanghai, China). Mice were bred and maintained in the Animal Resource Centre of the Faculty of Medicine, Fudan University. Mice had free access to food and water in a room with an ambient temperature of 22 ± 2°C and a 12:12-h light/dark cycle. All animal procedures were performed in strict accordance with the guidelines of the Institutional Animal Care and Use Committee of Fudan University. The ethics approval has been obtained from Experimental Animal Department of Fudan University.

### Reagents

Estradiol benzoate and progesterone were purchased from Aladdin (Shanghai, China). For animal experiments, estradiol benzoate and progesterone were dissolved in sesame oil. Anti-OXTR Ab (#ab217212, 1:300) was purchased from Abcam (Cambridge, U.K.). Anti-β-actin Ab (#BM0627, 1:4000) was purchased from Boster (Pleasanton, CA, U.S.A.). Anti-NLRP3 Ab (#AG-20B-0014-C100, 1:1000) was purchased from AdipoGen (San Diego, CA, U.S.A.). Anti-caspase-1 Ab (#06-503-I, 1:500) and anti-glial fibrillary acidic protein (GFAP) Ab (#MAB360, 1:500) were purchased from Millipore (Billerica, MA, U.S.A.). Anti-IL-1β Ab (#13767, 1:500) was purchased from Sigma (St. Louis, MO, U.S.A.). Anti-ASC Ab (#SC-22514-R, 1:500) was purchased from Santa Cruz (Dallas, Texas, U.S.A.). Pentobarbital sodium was purchased from Huayehuanyu (Beijing, China).

### Mouse model of PPD

Three-month-old female C57BL6J mice were made PPD model induced by HSP [27]. They were ovariectomized bilaterally using aseptic techniques under 1% pentobarbital sodium anesthesia (60 mg/kg, i.p.) and left for 7 days. The ovariectomized mice were injected hypodermically (i.h.) with estradiol benzoate (20 μg/kg) and progesterone (32 mg/kg) dissolved in sesame oil once a day for 16 consecutive days. Then progesterone was withdrawn and a high dose of estradiol benzoate (400 μg/kg) was administrated for further 7 days. Control mice were sham-operated and received sesame oil only. Three days after the last injection, behavioral evaluations were carried out. Mice were intraperitoneally injected 1% pentobarbital sodium (60 mg/kg) and then killed by quick cervical vertebra dislocation.

### Behavioral evaluations

#### Sucrose preference test

Sucrose preference was measured prior to ovariectomy and after the hormone injection, on day 0 and 33. Sucrose preference test (SPT) was performed as described previously [28]. After depriving of water for 12 h, mice were given the choice to drink from two bottles containing 1% sucrose solution or tap water, respectively, for 10 h. The positions of the bottles were switched after 5 h to prevent side preference in drinking behavior. The consumption of tap water and sucrose solution was estimated simultaneously in control and PPD groups by weighing the bottles. The preference for sucrose was calculated as a percentage of the consumed sucrose solution relative to the total amount of liquid intake.

#### Tail suspension test

Tail suspension test (TST) was performed as described previously [28]. Mice tails were wrapped with tape and fixed upside down on the hook. The immobility time of each mouse was recorded for a 6-min period. Mice were considered immobile only when they hung passively and completely motionless. The time of immobility during the last 4 min was measured with TailSuspScan (Clever Sys).

#### Forced swim test

Forced swim test (FST) was performed as described previously [28]. Mice were individually forced to swim in an open cylindrical container (25 cm in height and 10 cm in diameter) filled with water at room temperature (approximately 22 ± 1°C) to the depth of 14 cm for 6 min. Immobility was defined when the mouse floated in an upright position and made only small movements to keep its head above water for the requirement of respiration. The duration of immobility was recorded during the last 4 min by TailSuspScan (Clever Sys).

### Nissl staining

The brain slides were soaked in CV solution containing 0.1 g Cresyl Violet, 99 ml H_2_O and 1% acetic acid (1 ml) for 30 min at room temperature, then slides were dehydrated with alcohol and xylene. The slices were observed under stereomicroscope (Olympus).

### ELISA

Serum was assayed for oxytocin with ELISA kits from R&D Systems according to the manufacturer’s instructions. We set eight tubes of standard products with concentrations of 1000, 500, 250, 125, 62.5, 31.25, 15.625, 0 pg/ml as the abscissa, and the OD value as the ordinate. Then we drew a standard curve. We diluted the samples at a ratio of 1:2 and detected their OD values. According to the standard curve line, we found the corresponding oxytocin contents on the graph, and then multiply the dilution factor of 2.

### Real time quantitative-and reverse transcription-PCR

Total RNA was extracted from brain tissues using TRIzol reagent (Invitrogen, Carlsbad, CA, U.S.A.). Reverse transcription (RT) of total RNA was carried out using TaKaRa Master Mix (TaKaRa, Japan). The primers were purchased and validated from Generay (Shanghai, China). Real-time PCR was carried out using SYBR Green Master Mix (Applied Biosystems) in a StepOnePlus instrument (Applied Biosystems). The primers used for qPCR were as follows:

OXTR (F): CTCCCACCTATTTCTACTACC
OXTR (R): TCATTTCCCACTCCTTGTC
ENSMUSG00000090031 (F): CTGATGTTTGCCATAAAGAG
ENSMUSG00000090031 (R): AGTTAGGGAAGACAATGAAG
ENSMUSG00000087563 (F): GCCGTGATCTTGGGTTTG
ENSMUSG00000087563 (R): GCGACGATCTCGACTTTG
ENSMUSG00000104674 (F): CCCTTCAACTCCTTGGGTCC
ENSMUSG00000104674 (R): CCCAGGCTGGTGATTTCAGT
ENSMUSG00000109754 (F): TAGGCAAGAACTTCACGGTAG
ENSMUSG00000109754 (R): CTCTTTGTATGCCTGCGAATC
ENSMUSG00000045238 (F): TCGCATCAGTGCTGTGAAGT
ENSMUSG00000045238 (R): CGTCTTTCACGTGGATCCCT
GAPDH (F): CTGCCCAGAACATCATCC
GAPDH (R): CTCAGATGCCTGCTTCAC

### Western blotting analysis

Western blotting analysis was performed as described previously [29]. Brain tissues or cells were lysed in the buffer (Bio-Rad). Proteins were separated by SDS/PAGE using polyacrylamide TGX gels (Bio-Rad, Hercules, CA, U.S.A.) and then transferred to polyvinylidene difluoride (PVDF) membranes. After blocking, membranes were incubated with various specific primary antibodies as described above in TBST at 4°C overnight. Membranes were washed and incubated in corresponding secondary antibodies (1:1000, KPL) for 1 h at room temperature. Proteins were visualized and detected by enhanced chemiluminescence reagents (Pierce, Thermo Fisher Scientific) and analyzed with ImageQuant™ LAS 4000 imaging system (GE Healthcare, Pittsburgh, PA, U.S.A.).

### Immunocytochemical staining

Brain slides or astrocytes were rinsed with 0.1 M PBS and fixed with 4% paraformaldehyde, followed by block with PBS containing 5% bovine serum albumin, then incubated with the primary antibody (anti-GFAP Ab or anti-NLRP3 Ab) at 4°C overnight. After washing, brain slides or cells were exposed to fluorescent secondary antibody for 1 h at room temperature. After washing and treatment with DAPI (Life), cells were observed under stereomicroscope (Olympus, Tokyo, Japan).

### Transcriptome sequencing and bioinformatics analysis

The hippocampus was obtained for transcriptome sequencing using Illumina Truseq™ RNA Sample Prep Kit and bioinformatics analysis by Ucdom (Shanghai, China).

### Hippocampal primary astrocyte cultures and cell transfection

Mouse primary astrocyte cultures were conducted as described previously [29]. Plasmid of Gm14205, siRNA targeting OXTR or negative control (NC) siRNA (Jima, Shanghai, China) were transfected in primary astrocytes using Lipofectamine 3000 reagent (Invitrogen, Life Technologies) according to the manufacturer’s instructions. After 72 h, cells were collected for experiments.

### Statistical analysis

Data were presented as mean ± SEM. The significance of difference was determined by Student’s *t* test and two-factor analysis of variance. Difference was considered significant at *P*<0.05.

## Results

### HSP-induced PPD model mice exhibit depressive-like behaviors

**Figure 1:**
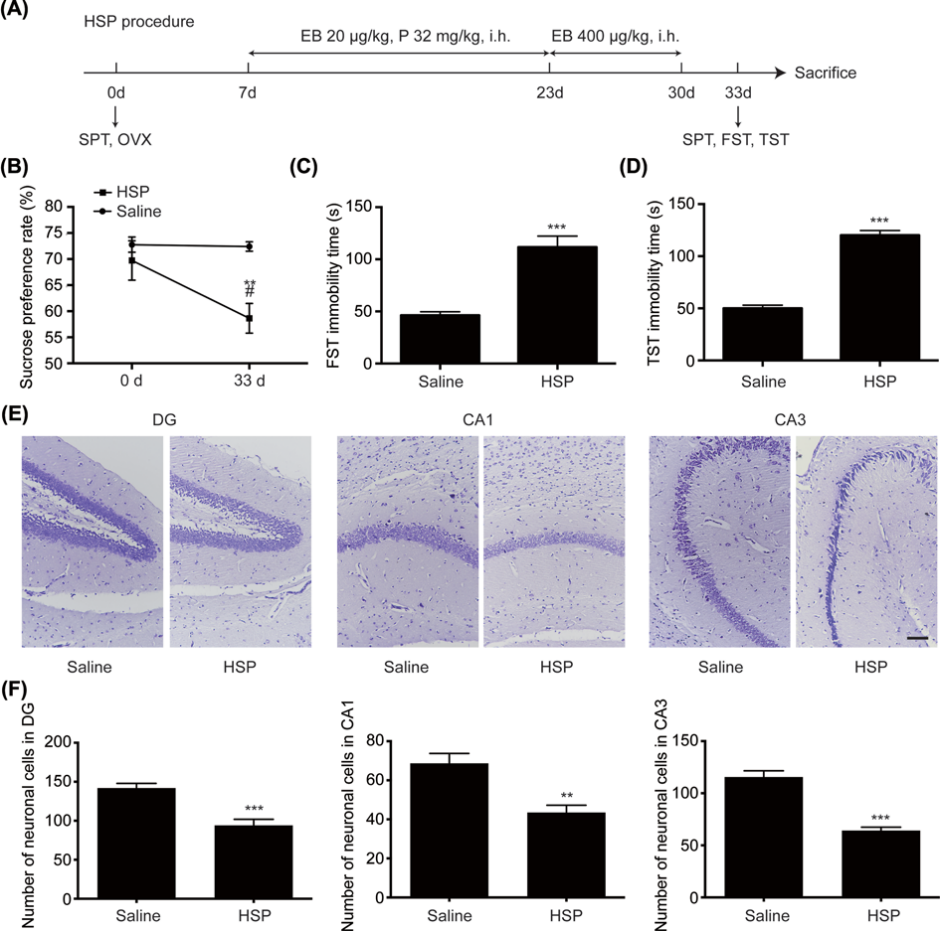
Behavioral evaluations of PPD model mice (**A**) The schedule of HSP-induced PPD mouse model and behavioral tests. Female C57BL6J mice were ovariectomized bilaterally and left for 7 days. The ovariectomized mice were i.h. with estradiol benzoate (20 μg/kg) and progesterone (32 mg/kg) once a day for 16 consecutive days. Then progesterone was withdrawn and a high dose of estradiol benzoate (400 μg/kg) was administrated for further 7 days. (**B**) Sucrose preference rate of SPT. (**C**) Immobility time of FST. (**D**) Immobility time of TST. (**E**) Nissl staining of DG, CA1, CA3 regions. (**F**) Counting of neuronal cells in DG, CA1, CA3 regions. Scale bar represents 100 μm. **P<0.01, ***P<0.001 vs Corresponding saline group, ^#^*P*<0.01 vs HSP 0 day group. Values are means ± SEM. Data are representative of at least five independent experiments. Abbreviation: OVX, ovariectomy.

The ovariectomized mice were subcutaneously injected with estradiol benzoate and progesterone to prepare PPD mouse model. SPT, FST, and TST were used for the evaluation of depressive-like behaviors, and the whole procedure is shown in Figure 1A. At the beginning of SPT, there was no significant difference in sucrose preference between two groups, while the sucrose preference rate of HSP group decreased by 11.10 ± 3.441% (Interaction F(1, 32) = 5.423, *P*=0.0263; Model F(1, 32) = 6.149, *P*=0.0186; HSP F(1, 32) = 13.28, *P*=0.0009) after modeling (Figure 1B). As shown in FST, the immobility time of HSP group increased by 65.47 ± 9.647 s (two-tailed, *t* = 6.786, df = 10, *P*<0.001) compared with control group after modeling (Figure 1C). Similarly, the immobility time of HSP group increased by 70.06 ± 5.090 s (two-tailed, *t* = 13.76, df = 10, *P*<0.001) in TST (Figure 1D). The Nissl staining results showed that the granule cells in the DG region and the vertebral cells in CA1 and CA3 regions of HSP group were loosely arranged (Figure 1E). The cell bodies became smaller, the nucleoli contracted, and the cells number decreased (Figure 1F) (DG: two-tailed, *t* = 4.802, df = 10, *P*<0.001; CA1: two-tailed, *t* = 3.968, df = 10, *P*=0.0027; CA3: two-tailed, *t* = 7.482 df = 10, *P*<0.001). The results above suggested that the establishment of PPD model was successful.

### The protein level of OXTR in hippocampus of PPD model mice was down-regulated

**Figure 2:**
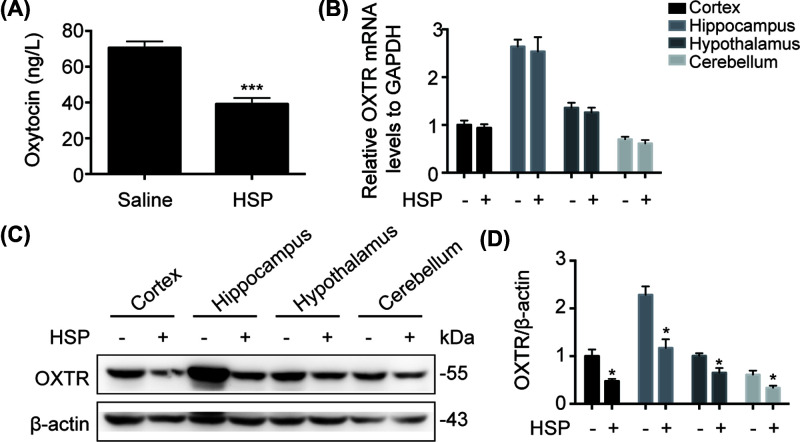
Expressions of oxytocin and OXTR in PPD model mice (**A**) Serum of mice was collected and oxytocin was detected by ELISA. (**B**) mRNA levels of OXTR in hippocampus, cortex, hypothalamus, cerebellum was analyzed by qRT-PCR. (**C**) OXTR expressed in hippocampus, cortex, hypothalamus, cerebellum of mice was analyzed by immunoblotting. (**D**) Densitometric analysis of OXTR. *P<0.05, ***P<0.001 vs Corresponding saline group. Values are means ± SEM. Data are representative of at least three independent experiments.

To explore the pathological mechanism of PPD, we detected the levels of oxytocin in serum of mice. As shown in Figure 2A, oxytocin was down-regulated in HSP group (two-tailed, *t* = 5.789, df = 30, *P*<0.001), suggesting the correlation between oxytocin and PPD. Then we respectively collected cortex, hippocampus, hypothalamus, cerebellum of mice and detected the expression of OXTR by real time-PCR and Western blot. As shown in Figure 2B, the mRNA level of OXTR was unchanged after modeling (cortex *P*=0.601; hippocampus *P*=0.7616; hypothalamus *P*=0.5058; cerebellum *P*=0.3416). The protein levels of OXTR in the four brain regions were all down-regulated (cortex *P*=0.0237; hippocampus *P*=0.012; hypothalamus *P*=0.0333; cerebellum *P*=0.0456) (Figure 2C,D). Notably, OXTR expressed in hippocampus was higher than that expressed in other brain regions, suggesting that hippocampus may be most closely related to PPD.

### NLRP3 inflammasome was activated in hippocampus of PPD model mice

**Figure 3:**
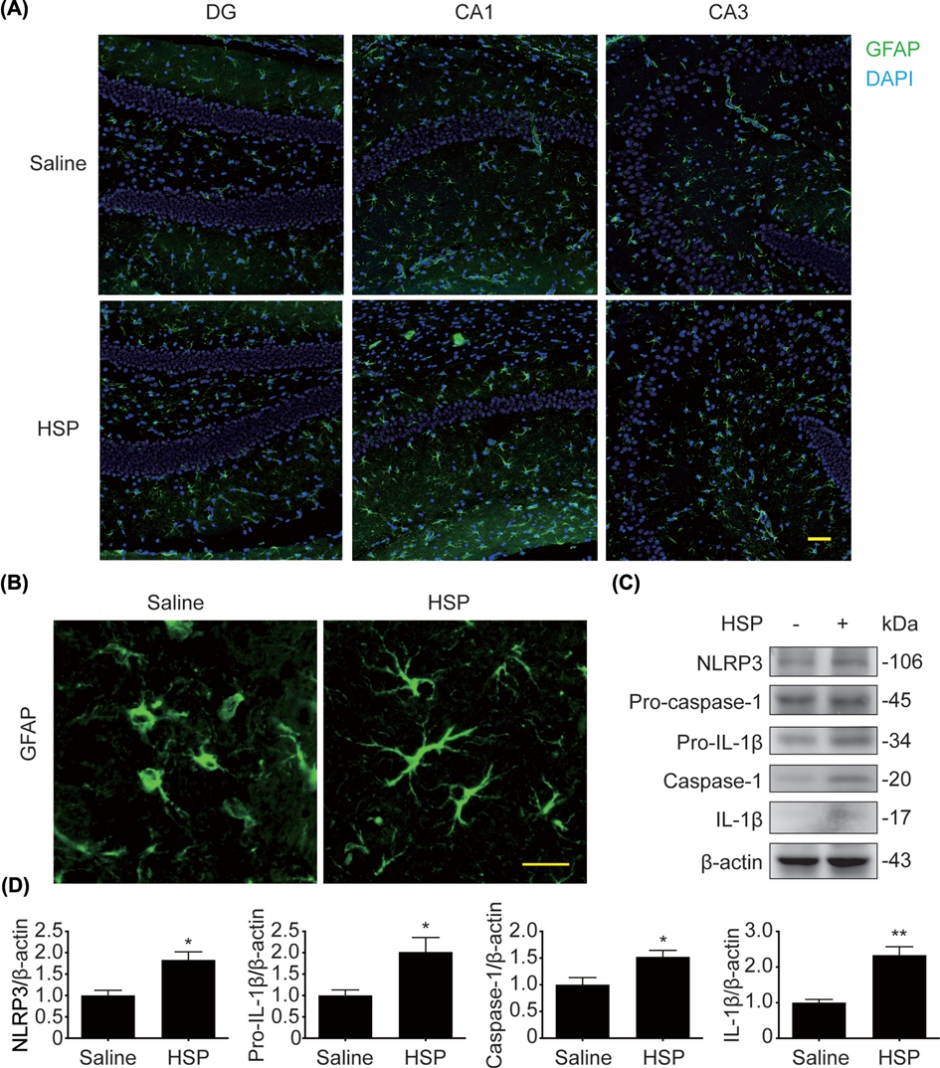
Astrocytes and NLRP3 inflammasome were activated in hippocampus of PPD model mice (**A,B**) GFAP^+^ cells in hippocampus were detected by immunofluorescence. GFAP^+^ cells were marked by green fluorescence. (**C**) NLRP3, pro-caspase-1, caspase-1, pro-IL-1β and IL-1β expressed in hippocampus were analyzed by immunoblotting. (**D**) Densitometric analysis of NLRP3, caspase-1, pro-IL-1β and IL-1β. Scale bar represents 50 μm. *P<0.05, **P<0.01 vs saline group. Values are means ± SEM. Data are representative of at least three independent experiments.

Considering that the mRNA level of OXTR in PPD model mice is not significantly reduced while the hippocampal neurons are remarkably lost, we speculate that non-neuronal cells which express OXTR are involved in the pathological process of PPD. Neuroinflammation is a common feature of many neurodegenerative diseases like depression [30]. It is mainly caused by activated glial cells and manifests as the secretion of inflammatory cytokines. Astrocytes are the most abundant glial cells in the brain, and they play a pivotal role in regulating inflammatory response in a variety of neurodegenerative diseases [31]. In order to investigate whether they are related to PPD, we detect the GFAP, a marker of astrocytes, by immunofluorescence. As shown in Figure 3A, the number of GFAP^+^ cells in the hippocampal DG, CA1, and CA3 regions of model mice did not change significantly compared with the saline group. And we found that the astrocytic protrusions in the hippocampal region of PPD model mice were long and thin, showing an active state (Figure 3B). Furthermore, we observed that NLRP3 inflammasome (two-tailed, *t* = 3.667, df = 4, *P*=0.0215) was activated and the proinflammatory cytokine interleukin-1β (IL-1β) (two-tailed, *t* = 5.251, df = 4, *P*=0.0063) was secreted in hippocampus of PPD model mice (Figure 3C,D).

### Non-coding RNA expression profiling in hippocampus of mice

**Figure 4:**
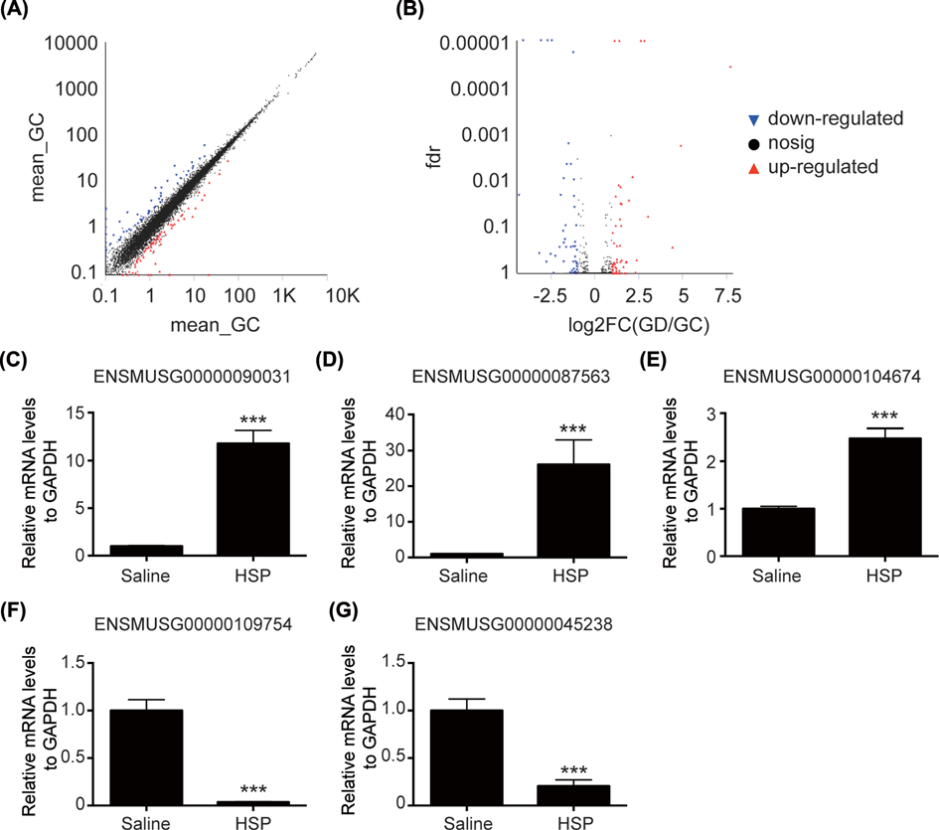
Validation of differentially expressed lncRNAs by qRT-PCR after RNA-seq (**A**) Scatter plot and (**B**) volcano plot of the differentially expressed genes. The transverse and vertical coordinates are logarithmized. Each point represents a specific gene or transcript. The red dots indicate genes that are significantly up-regulated, the blue dots indicate genes that are significantly down-regulated, and the black dots are non-significantly differentially expressed genes. (**C**–**G**) Differentially expressed lncRNAs were detected by qRT-PCR. ***P<0.001 vs Corresponding saline group. Values are means ± SEM. Data are representative of at least three independent experiments.

Considering the protein level of OXTR in hippocampus of PPD model mice was down-regulated while the mRNA level of it in hippocampus was unchanged, it suggested that post-transcriptional mechanism played a role in the pathological process. Therefore, differentially expressed non-coding RNAs in hippocampus were identified using transcriptome sequencing. As shown in Supplementary Table, we found 27 differentially expressed genes (|log2FC|>1 and FDR < 0.05. FC, fold change; FDR, false discovery rate), including five differentially expressed lncRNAs. Among the five differentially expressed lncRNAs (Table 1), three were up-regulated (ENSMUSG00000090031, ENSMUSG00000087563, ENSMUSG00000104674) and two were down-regulated (ENSMUSG00000109754, ENSMUSG00000045238). The overall distribution of the differential genes can be inferred by visualizing the scatter plot (Figure 4A) and volcano plot (Figure 4B). To validate the transcriptome sequencing results, we used qRT-PCR to detect the differentially expressed lncRNAs. As shown in Figure 4C–G (*P*<0.001), the qRT-PCR results were concordant with the transcriptome sequencing data.

**Table 1:** Differentially expressed lncRNAs between control and PPD groups

Id	Type	Name	log2FC (GD/GC)	*P*-value	FDR	Regulation
ENSMUSG00000090031	lncRNA	4732440D04Rik	2.641215914	3.16E-12	9.03E-09	up
ENSMUSG00000109754	lncRNA	Gm39214	−2.672129766	5.69E-10	1.22E-06	down
ENSMUSG00000087563	lncRNA	Gm14205	7.723717533	2.09E-08	3.59E-05	up
ENSMUSG00000045238	lncRNA	A730035I17Rik	−1.926173533	2.94E-05	0.021068064	down
ENSMUSG00000104674	lncRNA	Gm42756	1.531618517	5.83E-05	0.032296743	up

To avoid false positive errors, multiple test correction method was used to correct the significant *P*-value obtained from the original hypothesis test, and finally FDR was used as key index for screening differentially expressed genes. FDR < 0.05.

### Gene Ontology and KEGG analysis of differentially expressed genes

**Figure 5:**
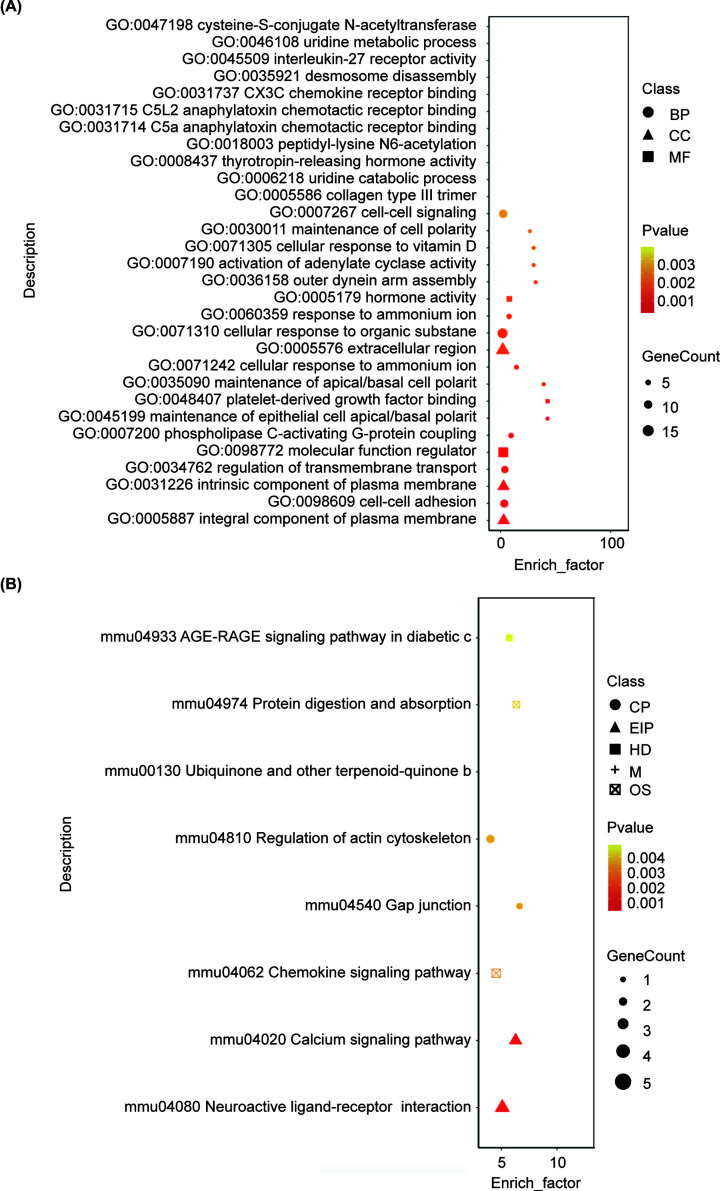
GO and KEGG enrichment analyses (**A**) GO enrichment results in scatter diagram, the top 30 GO categories are listed. All the genes/transcripts were selected as background lists, and the differentially expressed genes/transcripts were selected as candidate lists from background lists. Fisher’s exact test was used. In order to control the calculated false positive rate, four multiple tests (Bonferroni, Holm, Sidak, and FDR) were used to correct the *P*-value. Generally, when the corrected *P*-value (*P*_fdr) is less than 0.05, it is considered that the GO function is significantly enriched. (**B**) Top eight enriched signaling pathways from KEGG analysis. Fisher exact test is used for calculation. In order to control the false positive rate, BH (FDR) method was used to carry out multiple tests. Corrected *P*-value was defined as a KEGG pathway with a threshold of 0.05.

After the transcriptome sequencing, we performed the bioinformatics analysis. Using Gene Ontology (GO) database, genes can be classified according to the biological processes (BP) in which they participate, the cellular components (CCs) that make up the cells, and the molecular functions (MF) that are achieved. The top 20 enriched terms of differentially expressed genes in the BP, CC, and MF terms are presented in Figure 5A, including extracellular region, cellular response to organic substance, MF regulator, cell adhesion, biological adhesion, etc. In terms of the Kyoto Encyclopedia of Genes and Genomes (KEGG) analysis of our study, a total of 108 signaling pathways were enriched, and the top 8 are listed in Figure 5B. Among those pathways, ‘neuroactive ligand–receptor interaction’ was enriched the most, followed by calcium signaling pathway, chemokine signaling pathway, regulation of action cytoskeleton, etc. Notably, OXTR signaling is closely associated with the mostly enriched pathway ‘neuroactive ligand–receptor interaction’. Therefore, we speculated that lncRNA regulates PPD by targeting OXTR.

### LncRNA Gm14205 negatively regulated OXTR and activated NLRP3 inflammasome

**Figure 6:**
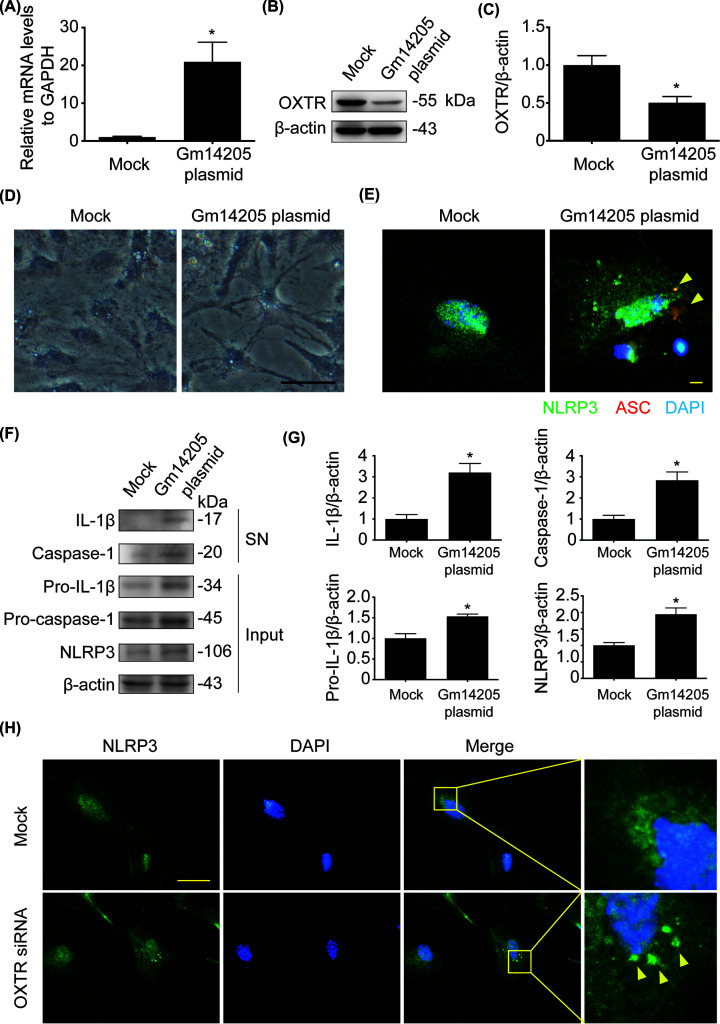
The regulation of lncRNA Gm14205 to OXTR and NLRP3 (**A**) Transfected lncRNA Gm14205 were measured by qRT-PCR. (**B**) OXTR expressed in primary astrocytes was analyzed by immunoblotting. After transfecting lncRNA Gm14205 in primary astrocytes, the protein level of OXTR was detected. (**C**) Densitometric analysis of OXTR. (**D**) Cell morphology was observed under the bright field. (**E**) NLRP3 and ASC were detected by immunofluorescence in primary astrocytes transfected with Gm14205 plasmid or not. NLRP3 was marked by green fluorescence and ASC was marked by red fluorescence. (**F**) NLRP3, pro-caspase-1, caspase-1, pro-IL-1β and IL-1β expressed in astrocytes were analyzed by immunoblotting. (**G**) Densitometric analysis of NLRP3, caspase-1, pro-IL-1β and IL-1β. (**H**) NLRP3 in primary astrocytes was detected by immunofluorescence. After transfecting OXTR siRNA in primary astrocytes, NLRP3 was marked by green fluorescence. Scale bar represents 20 μm. *P<0.05, **P<0.01 vs Mock group. Values are means ± SEM. Data are representative of at least three independent experiments.

Among the five differentially expressed lncRNAs, the expression of ENSMUSGC00000087563 (Gm14205) varied the most (RNA-seq: |log2FC| = 7.7237, FDR = 3.59E-05; qRT-PCR: 26.1-fold, *P*=0.0007). Thus, we cultured mouse hippocampal primary astrocytes and explored the effect of lncRNA Gm14205 on regulating OXTR. As shown in Figure 6A–C, after transfecting plasmid which carries lncRNA Gm14205 in liposome (qRT-PCR: 20.97-fold, *P*=0.0181), the protein level of OXTR was down-regulated (two-tailed, *t* = 3.293, df = 4, *P*=0.0301). It suggested that lncRNA Gm14205 targets OXTR. Cell morphology was observed under the bright field, and the protrusions of astrocytes transfected with lncRNA Gm14205 plasmid were long and thin (Figure 6D). The immunofluorescence (Figure 6E) showed that NLRP3 was up-regulated and ASC speck was formated, suggesting that NLRP3 inflammasome was activated. We collected and extracted the proteins in the cell cytoplasm and supernatant respectively, and detected the levels of NLRP3, IL-1β/pro-IL-1β, caspase-1/pro-caspase-1. The results (Figure 6F,G) showed that the secretion of IL-1β (two-tailed, *t* = 4.571, df = 4, *P*=0.0103) and caspase-1 (two-tailed, *t* = 4.232, df = 4, *P*=0.0133) are increased in the cell supernatant, and the level of NLRP3 (two-tailed, t = 4.441, df = 4, *P*=0.0113), and pro-IL-1β (Two-tailed, t = 4.092, df = 4, *P*=0.0150) in the cytoplasm was up-regulated, which also suggesting that NLRP3 inflammasome was activated. Furthermore, we delineated the effect of OXTR on NLRP3 inflammasome activation by interfering OXTR. Immunofluorescence result showed that knockdown of OXTR (Figure 6H) activated NLRP3 inflammasome. These results indicated that lncRNA Gm14205-OXTR-NLRP3 axis may be a possible pathological mechanism in PPD.

## Discussion

Modern women are under great pressure of work and life, and the incidence of PPD is also on the rise [11]. PPD not only seriously damages women’s physical and mental health, but also brings harmful impact on the healthy growth of infants, family harmony, and social stability [32]. Its pathological mechanisms have not been fully elucidated yet, and researches usually involve monoamine transmitter deficiency, neuroendocrine disorder, neurotrophic factor endocrine reduction, oxidative stress, neuroinflammation hypothesis, etc [3,33]. However, most of the studies focus on depression itself, ignoring the important attribute of ‘postpartum’. Thus, it reminds us that we should search for some targets that relate to the puerperium. Recently, Zulresso [34,35] (brexanolone injection, an allosteric regulator that can simultaneously act on synaptic and extrasynaptic GABA_A_ receptors), developed by Sage Therapeutics Biopharmaceutical Company, has got the approval of the Food and Drug Administration (FDA) for the first drug specializing in treatment of PPD. It reflects from the side that the research field on PPD still has a very broad space. Oxytocin and OXTR are closely related to childbirth and lactation, and they also play important roles in the treatment of some psychiatric diseases [36]. Previous study has shown that injection of exogenous oxytocin into the paraventricular nucleus of hypothalamus can improve the depression-like behavior of PPD model rats and play an anti-PPD role [37]. Our study showed that oxytocin was down-regulated in HSP group. The result is consistent with previous report. We also found the protein level of OXTR in hippocampus of PPD model mice was down-regulated. These findings imply that OXTR may be a promising target for the therapy of PPD.

Astrocytes have a basic physiological function in forming borders to restrict access of leukocyte into brain parenchyma [31]. They also provide energy sources for neurons, modulate synaptic activity, and regulate extracellular glutamate levels [38]. Astrocytes have been reported to be associated with many kinds of neurodegenerative disease like depression [39,40]. Previous studies showed that neuroinflammation reactions occur in multiple brain regions in animal models with depression [41,42]. Our study also revealed that astrocytes and NLRP3 inflammasome in hippocampus of PPD model mice were activated and caused the subsequent the secretion of proinflammatory cytokine IL-1β. In addition, previous animal models of depression [43,44] showed that the number and density of astrocytes are significantly decreased compared with normal cases. Interestingly, in our present study, astrocytes just activate rather than obviously lose in PPD model mice. Perhaps it revealed the differences of pathology between PPD and ordinary depression. The in-depth mechanism is currently unknown and deserves further explorations.

It has been reported that hypermethylation and low expression of OXTR may play an important role in the etiology of PPD susceptible phenotypes [20–22], suggesting that their post-transcriptional mechanisms may regulate the occurrence of PPD. Our study showed that the protein level of OXTR was decreased while the mRNA level was unchanged, suggesting a potential post-transcriptional mechanism as well. Post-transcriptional regulation refers to the regulation of gene expression after RNA transcription and is one of the characteristics of eukaryotic gene expression [45,46]. Studies have shown that lncRNA may participate in the pathophysiological process of depression and regulate DNA transcription and chromosome remodeling [26,47]. It has been reported that the expression of lncRNA in peripheral blood mononuclear cells of depressive patients is significantly down-regulated. However, the regulatory effect of lncRNA on PPD, such a special type of depression, has not been reported yet. In the present study, we identified five lncRNAs related to PPD by transcriptome sequencing, including three up-regulated (ENSMUSG00000090031, ENSMUSG00000087563, ENSMUSG00000104674) and two down-regulated (ENSMUSG00000109754, ENSMUSG00000045238). The five lncRNAs are associated with the signaling pathway of OXTR according to the bioinformatics analysis. Furthermore, we found that overexpression of lncRNA Gm14205, a lncRNA with the biggest variation among the five differentially expressed lncRNAs, inhibited the protein level of OXTR in hippocampal primary astrocytes, and knockdown of OXTR-activated NLRP3 inflammasome. The results above suggest that lncRNA Gm14205 may be a crucial biological target in PPD.

**Figure 7:**
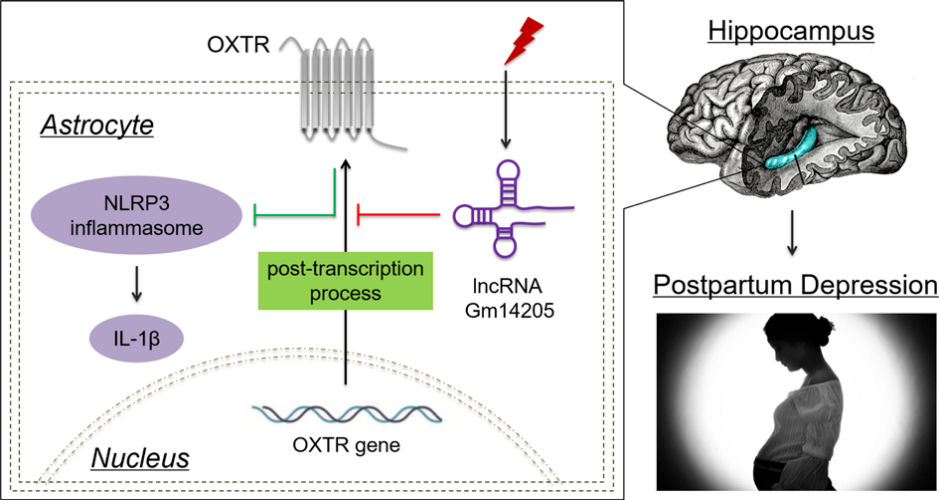
Schematic diagram of the anti-inflammasome effect of lncRNA Gm14205 targeting OXTR A proposed model for how lncRNA Gm14205 regulates OXTR and modulates NLRP3 inflammasome activation in hippocampal astrocytes in PPD.

In summary, our study reveals a novel function of lncRNA Gm14205–OXTR–NLRP3 axis in the pathology of PPD as shown in Figure 7. However, there are further experiments worthy doing to enrich our studies: (1) our study shows that astrocytic OXTR in hippocampus may play an important role in PPD. However, we do not know whether OXTR in other kinds of glial cells like microglia also has the function. Microglia are the most sensitive cells to inflammasome responses in the CNS, and it is very meaningful to check for microglial inflammasome activation. Further studies using astrocytic and microglial OXTR conditional knockout mice (OXTRloxp/loxp; GFAP-cre; OXTR loxp/loxp; Iba1-cre) will provide more conclusive evidence. (2) We speculate that lncRNA Gm14205 inhibited OXTR possibly through a post-transcriptional regulation, but the precise mechanism remains unknown. We did not investigate that the reduction in OXTR may occur at the protein level, instead of the mRNA level. The ubiquitination degradation of OXTR protein may also be involved in the pathological process. Further explorations will be done to confirm and enrich the study. (3) Except lncRNA Gm14205, other four differentially expressed lncRNAs may also play roles in PPD, and further studies deserve to be done to expand our researches. Collectively, these results illustrate that OXTR has protective effects by suppressing NLRP3 inflammasome activation and provide a new strategy for targeting lncRNA Gm14205 in the pathogenesis of PPD. It will help us accumulate academic basis for clinical diagnosis and drug development, promoting the translation from basic research to clinical application.

## Supplementary Material

Supplementary Table

## Abbreviations

Ab: anti-body
ASC: Apoptosis-associated speck-like protein containing a CARD
BP: biological process
CV: cresyl violet
DAPI: 4′,6-diamidino-2-phenylindole
FC: fold change
FDR: false discovery rate
FST: forced swim test
GABA: gamma-aminobutyric acid
GFAP: glial fibrillary acidic protein
GO: Gene Ontology
HSP: hormone-simulated pregnancy
i.p.: intra-peritonelly
IL-1β: interleukin-1β
lncRNA: long non-coding RNA
MF: molecular function
NLRP3: Nod-like receptor protein 3
OD: optical density
OXTR: oxytocin receptor
PPD: postpartum depression
SPT: sucrose preference test
TBST: Tris Buffered saline Tween
TST: tail suspension test
