# Supplementary material for: LncRNA Gm14205 induces astrocytic NLRP3 inflammasome activation via inhibiting oxytocin receptor in postpartum depression
Source: Biosci Rep. 2020 Aug 7;40(8):BSR20200672. doi: 10.1042/BSR20200672 (PMC7414522; doi:10.1042/BSR20200672)
Supplement: Supplementary Table [file BSR-2020-0672_supp.pdf]

Supplementary Table. Differentially expressed genes between control and PPD groups

id	type	name	log2FC(GD/GC)	pval	fdr	regulate
ENSMUSG00000054074	mRNA	Skida1	-4.059131148	4.22E-47	7.25E-43	down
ENSMUSG00000053004	mRNA	Hrh1	2.816998418	6.35E-16	5.45E-12	up
ENSMUSG00000038178	mRNA	Slc43a2	1.403245835	1.44E-15	8.24E-12	up
ENSMUSG00000032484	mRNA	Ngp	-3.032195491	7.20E-13	3.09E-09	down
ENSMUSG00000103677	mRNA	Pcdhga4	-2.444296501	1.31E-12	4.50E-09	down
ENSMUSG00000090031	lncRNA	4732440D04Rik	2.641215914	3.16E-12	9.03E-09	up
ENSMUSG00000038145	mRNA	Snrk	1.131226533	7.87E-12	1.93E-08	up
ENSMUSG00000109754	lncRNA	Gm39214	-2.672129766	5.69E-10	1.22E-06	down
ENSMUSG00000066735	mRNA	Vkorc1l1	-1.225006946	9.43E-09	1.80E-05	down
ENSMUSG00000087563	lncRNA	Gm14205	7.723717533	2.09E-08	3.59E-05	up
ENSMUSG00000045573	mRNA	Penk	-1.498798792	1.15E-06	0.001646706	down
ENSMUSG00000094695	mRNA	Gm21953	4.894733705	1.36E-06	0.001801382	up
ENSMUSG00000027852	mRNA	Nras	-1.589186742	4.09E-06	0.004579247	down
ENSMUSG00000041534	mRNA	Rbp3	-1.341444052	4.27E-06	0.004579247	down
ENSMUSG00000005892	mRNA	Trh	2.139362423	8.39E-06	0.008470327	up
ENSMUSG00000049421	mRNA	Zfp260	-1.758463962	1.02E-05	0.009235512	down
ENSMUSG00000053119	mRNA	Chmp3	-1.164333628	1.15E-05	0.00984957	down
ENSMUSG00000093989	mRNA	Rnasek	1.358751263	1.56E-05	0.012731231	up
ENSMUSG00000035829	mRNA	Ppp1r26	1.48530434	1.79E-05	0.013958752	up
ENSMUSG00000029711	mRNA	Epo	-4.305005399	2.88E-05	0.021068064	down
ENSMUSG00000045238	lncRNA	A730035I17Rik	-1.926173533	2.94E-05	0.021068064	down
ENSMUSG00000075334	mRNA	Rprm	-1.171088864	3.36E-05	0.022167348	down
ENSMUSG00000049281	mRNA	Scn3b	1.19790544	3.36E-05	0.022167348	up
ENSMUSG00000042523	mRNA	Dnal1	1.9593073	4.40E-05	0.027001684	up
ENSMUSG00000038797	mRNA	Zscan2	1.485104121	5.47E-05	0.03132668	up
ENSMUSG00000104674	lncRNA	Gm42756	1.531618517	5.83E-05	0.032296743	up
ENSMUSG00000001119	mRNA	Col6a1	-1.139747859	7.57E-05	0.038251241	down

Supplementary Table. Differentially expressed genes between control and PPD groups

To avoid false positive errors, multiple test correction method was used to correct the significant

p-value obtained from the original hypothesis test, and finally false discovery rate (FDR) was used as key index for screening differentially expressed genes. FDR<0.05
